# Community Health Workers Can Provide Psychosocial Support to the People During COVID-19 and Beyond in Low- and Middle- Income Countries

**DOI:** 10.3389/fpubh.2021.666753

**Published:** 2021-06-22

**Authors:** Sabuj Kanti Mistry, Ben Harris-Roxas, Uday Narayan Yadav, Sadia Shabnam, Lal Bahadur Rawal, Mark F. Harris

**Affiliations:** ^1^BRAC James P Grant School of Public Health, BRAC University, Dhaka, Bangladesh; ^2^Centre for Primary Health Care and Equity, University of New South Wales, Sydney, NSW, Australia; ^3^Health Nutrition and Population Program, BRAC, Dhaka, Bangladesh; ^4^School of Health Medical and Applied Sciences, Central Queensland University, Sydney Campus, Rockhampton, QLD, Australia

**Keywords:** community health workers, psychosocial support, low- and middle- income countries, COVID-19, mental health

## Abstract

The COVID-19 pandemic has been the most challenging public health issue which not only affected the physical health of the global population but also aggravated the mental health conditions such as stress, anxiety, fear, depression and anger. While mental health services are seriously hampered amid this COVID-19 pandemic, health services, particularly those of Low- and Middle- Income Countries (LMICs) are looking for alternatives to provide psychosocial support to the people amid this COVID-19 and beyond. Community Health Workers (CHWs) are an integral part of the health systems in many LMICs and played significant roles such as health education, contact tracing, isolation and mobilization during past emergencies and amid COVID-19 in many LMICs. However, despite their potentials in providing psychosocial support to the people amid this COVID-19 pandemic, they have been underutilized in most health systems in LMICs. The CHWs can be effectively engaged to provide psychosocial support at the community level. Engaging them can also be cost-saving as they are already in place and may cost less compared to other health professionals. However, they need training and supervision and their safety and security needs to be protected during this COVID-19. While many LMICs have mental health policies but their enactment is limited due to the fragility of health systems and limited health care resources. CHWs can contribute in this regard and help to address the psychosocial vulnerabilities of affected population in LMICs during COVID-19 and beyond.

## Introduction

The COVID-19 pandemic has been the most challenging global public health issue so far in this century. With the notable exception of climate change (1), there have been no events that have affected nearly every person on the planet in the same way COVID has.

The disease continues to spread across the world, with over 85 million confirmed cases with over 1.8 million deaths up to 7th January 2021 (2). The advent of COVID-19 has not only seriously hampered the physical well-being of a vast majority of global people but also aggravated the mental health conditions of many people. The unplanned lockdown and isolation imposed due to COVID-19 and subsequent difficulty in accessing food, health care, medication, and psychological support have exacerbated anxiety, depression, post-traumatic stress disorder among people from all age group (3, 4).

Historically, the mental health and well-being of the population has been disrupted due to the large-scale outbreaks and pandemics. For example, during the H_1_N_1_ influenza virus outbreak in 2009, anxiety among the UK's general population increased by 10–30% (5). Similarly, during the severe acute respiratory syndrome (SARS) epidemic in 2002–2004, psychiatric morbidities, depression, and stress disorder increased (6). During the Ebola outbreak, in 2013–2016 in Guinea, Liberia, and Sierra Leone, the psychosocial well-being of the people was seriously hampered (7). It is not surprising that - stress, anxiety, depressive symptoms, insomnia, denial, anger, and fear are associated with the COVID-19, (8, 9) even amongst those who have not been infected.

Mental health services have also been seriously disrupted during the COVID-19 pandemic worldwide. A recent survey carried out in 130 countries reported that mental health services were seriously hampered during the pandemic (10). Several researchers pointed the challenges to mental health services during this pandemic and suggested for alternative ways to provide psychosocial support services (11). Telemedicine or online health services have been used to some extent as an alternative way to provide healthcare services (12). Yet, people from LMICs with limited access to the internet are largely unable to avail themselves of these services (13).

## Who are community health workers?

The World Health Organization (WHO) defines “community health worker” as an umbrella term which encompasses a variety of community health aides working in their own community. According to WHO, “community health workers should be members of the communities where they work, should be selected by the communities, should be answerable to the communities for their activities, should be supported by the health system but not necessarily a part of its organization, and have shorter training than professional workers” (14). The American Public Health Association (APHA) defines community health workers more expansively as, “a frontline public health worker who is a trusted member of and/or has an unusually close understanding of the community served. This trusting relationship enables the CHW to serve as a liaison/link/intermediary between health/social services and the community to facilitate access to services and improve the quality and cultural competence of service delivery” (15).

Globally, CHWs are considered as an integral part of the health care system in achieving universal health coverage for all individuals (16, 17). The most recent estimates suggest that there are around five million CHWs currently working worldwide. The WHO have forecasted a global shortage of 18 million trained health professionals by 2030 (18). CHWs have been shown to be a cost-saving way to complement the shortages of health professionals in implementing community-based health care programs, as well as representing a potentially scalable workforce (19). The concept of CHWs primarily evolved in LMICs during the 1970s (20). Further, in the Alma Ata declaration (1978), the WHO explicitly pointed to the importance of CHWs in providing effective primary health care (PHC), by ensuring access to basic health services for the underserved that address local health needs and engage the community (21). Over the last decade many CHW-led programs in LMICs have been restructured to delivering primary health care services for infectious diseases as well as services for prevention and management of non-communicable diseases (22). Some of the most well-known, large-scale and effective CHW programs in LMICs are *gentes Comunitários de Saúde* in Brazil, *Sasthya Sebika* and *Sasthya Kormis* of BRAC, Female Community Health Volunteer (FCHV) in Nepal, Lady Health Worker in Pakistan, and Accredited Social Health Activists (ASHAs) in India (20). There has also been growing attention in recent years regarding the potential contribution of CHWs in high-income countries in reducing health care inequalities as well (23, 24).

## Existing roles of CHWs

CHWs were trained and deployed throughout the world to address the crisis in healthcare workforce in the 1970s, especially for underserved communities whose healthcare needs were not addressed by the formal healthcare systems (25). The role of CHWs can be varied based on the need of the communities they served (26). CHWs play an important role in addressing health care inequalities by assisting people to access services offered by the formal health care system (22). Several studies have identified different roles of CHWs including health assessment (27) and facilitating treatment (28), health care navigation (29), health education (30), psychosocial support (31) and data collection (32). CHW interventions were previously focused mostly on maternal child health (33, 34), prevention of malnutrition (35) and control of infectious disease such as HIV/AIDS and tuberculosis (35, 36). In recent years, the potential to engage CHWs in the prevention and control of non-communicable diseases in countries of South and South East Asia also has been well-documented (37, 38). Recent research has demonstrated that CHWs are effective in improving the accessibility and acceptability of preventive care and primary health care and providing health education. In these roles they have reduced hospitalization and re-hospitalization particularly for people from disadvantaged communities (39).

## Roles of CHWs in emergencies

Despite strong evidence on the contribution of CHWs at primary health care and community levels, their potential in outbreaks may be under-utilized or overlooked. Several studies (40–42) conducted during previous as well as COVID-19 outbreak pointed out that CHWs were largely underutilized in emergency management and the emergency response failed due largely to not engaging them. While acknowledging the under-utilization of the CHWs at a broader scale in COVID-19 management, Haines et al. also called for training and engaging CHWs to support people in their own homes during this COVID-19 pandemic in UK (43). Peretz et al. also recommended use of CHWs to address the social determinants of health during the COVID-19 pandemic (44).

There is strong evidence supporting the role of CHWs in emergency situations such as humanitarian emergencies and infectious outbreaks when demand for health services increases. BRAC Bangladesh developed the concept of CHWs (*Sasthya Sebikas*) when there was a diarrhea epidemic in Bangladesh in the 1980s and CHWs played a pivotal role in educating people in terms of prevention and control of diarrhea (45).

It has also been demonstrated during the Ebola outbreak in Guinea, Sierra Leone, and Liberia that CHWs were effective in emergency management through the early identification of cases as well as in pandemic preparedness through proper communication in a culturally appropriate way, community education and mobilization, contribution to surveillance systems, and filling health service gaps (46). Likewise, CHWs enabled Nigeria to quickly treat and isolate cases, thereby helped eradicate the polio from the country (47).

## CHWs during COVID-19 and in psychosocial support

Establishment of a community centric care model of care with CHWs at the center can be effective in managing the COVID-19 pandemic as well as potential future episodes (48, 49). This community centric approach facilitates proactive community-wide services such as rapid testing, contact tracing, promoting isolation and quarantine which are considered crucial in slowing down the spread of COVID-19 (48, 50). CHWs can also be very crucial in providing psychosocial support to the people amid COVID-19 pandemic. In trials carried out in India (51), Uganda (52), Nepal (53) and Pakistan (54), 70% of those cared for by trained CHWs recovered from mental health problems. They have also helped to compensate for critical health workforce shortage during this pandemic, particularly in LMICs (49).

## The unrealised potential of CHWs in providing psychosocial support

**Figure 1:**
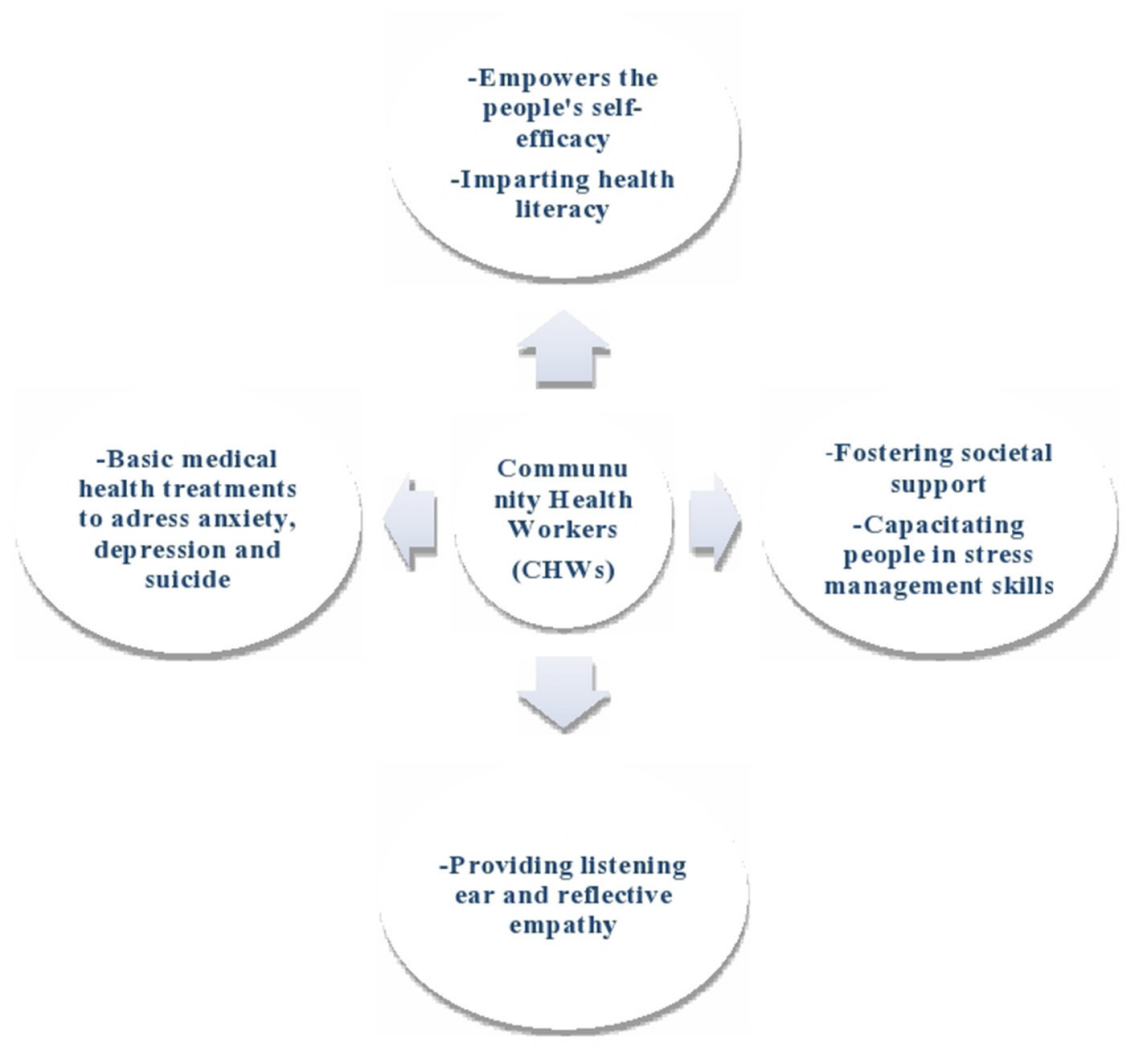
Potential roles of CHWs in providing psychosocial supports.

As CHWs are already in touch with the community as their trusted members, they can be engaged to provide psychosocial support to the people who were at increased need due to COVID-19 related stress, anxiety and depression (44, 55). For example, regular home visit is an integral component of the most CHW programs carried out in Bangladesh (56), India (57), Kenya (58), Brazil (59), Pakistan (60) and Nepal (61) (Table 1). Therefore, these CHWs can be trained to provide psychosocial support either face-to- or over telephone (44, 62). Although home visits are limited during this pandemic, CHWs can be the best option who can connect the people to nearby primary health care providers and mental health resources. Moreover, the ethos of CHWs is to empower and engage people in health promotion behaviors, enhance their health literacy in local languages, foster social support, and provide a listening ear (44). Adding to this, CHWs can provide basic mental health support to address stress, anxiety, and depression, which have risen during COVID-19 (63). Figure 1 summarizes potential roles for CHWs in providing psychosocial support.

**Table 1:** Overview of the major global CHW interventions.

**Name of CHW**	**Program**	**Country**	**Role**
Agentes Comunitários de Saúde (59)	Programa de Agentes Comunitários de Saúde (PACS)	Brazil	The major role of them is to undertake home visits to disseminate health information, provide essential health care services, and establish referral to primary care when required.
*Sasthya Sebika* and *Sasthya Kormi* (56)	BRAC Health Nutrition and Population Programme	Bangladesh	As part of BRAC Health initiatives SSs and SKs perform regular home visits to provide maternal and child health care services such as antenatal, natal and post-natal care services, essential health care services common ailments, health education and so forth.
FCH (61)	Female Community Health Volunteer (FCHV) program	Nepal	The primary roles of FHVs are health promotion, delivering health services such as polio campaign, family planning, referral to primary care, data collection etc. through community mobilization.
LHW (60)	Lady Health Worker Program (LHWP)	Pakistan	LHWs provides reproductive health services such as family planning, maternal and child health services to the doorstep of the people.
CHW (58)	Living Goods	Kenya	CHWs are assigned to a selected number of households and involved in case management of diarrhea, pneumonia, malaria, pregnancy and newborn care. They undertake regular home visits and sell some products to get some money.
ASHA worker (57)	Accredited Social Health Activists (ASHAs) program	India	Of many roles the ASHA workers play, the major initiatives include regular home visits to promote reproductive and child health care services to the doorstep of the marginalized people.

Incorporating CHWs in providing psychosocial support can be cost saving. They have already been demonstrated to be a less expensive alternative compared to other health professionals (64). On the other hand, little extra effort is needed to recruit or engage them as many of them are already employed casually or part time. For example, a study conducted by Fiedler et al. (65) in Honduras found that CHW programme cost only 11% of the facility-based care. Likewise, Datiko and Lindtjørn (66) found that involving Health Extension Workers (HEWs) in TB treatment was a cost-effective alternative to health facility delivery. Surprisingly, despite their potential to provide psychosocial support to the people at the community level in LMICs, particularly during this overwhelming situation, CHWs have been underutilized in this role (67, 68).

Cultural factors may stop people presenting with psychological conditions or mask them as somatised physical symptoms. CHWs are well-placed to address this because they understand the cultural belief and practices of their communities. However, they need specific training in how to address possible conflicts between cultural beliefs and western health care (69).

## Investment in CHWs' capacity building

CHWs need to be equipped with adequate training before they are engaged in providing psychosocial support. They need to be trained in assessment, communication skills, problem solving, professional responsibilities and boundaries, as well as stress and emotion management strategies. Previous research has demonstrated that short duration training for up to 2 weeks produced good results (56, 70, 71). For example, Barnett et al. (71) found that a 5-day training resulted in improved ability of the CHWs in identification and management of mental health issues among the patients. Alternative models of delivery such as e-learning may be of value in the context of COVID-19 (72). Digital technologies can be effectively employed to conduct training and provide supervision remotely (49, 73). Also, digital technology can be one of the less expensive ways to disseminate the mental health information as the number of people using phones and internet is increasing with an estimate of 84% in LMICs (74).

Management of CHWs requires sustainable support by and integration into local and national health systems, plans and policies. It also requires supportive supervision that solves problems and improves skills. To address most of the mental health problems at the community level, CHWs need to be trained on providing psychosocial support for basic mental illnesses such as anxiety, fear, and depression. One study (75) also reported that poor supervision led to compromised CHW performance. Also, they need to be well-trained in navigation for the people with moderate or severe mental health illness to the nearest health service.

## Safety and security of CHWs

While the importance of CHWs' engagement in effective COVID-19 management has been well-documented, it is also essential that several precautions are taken before scaling up CHWs roles in COVID 19 management. CHWs should be provided with appropriate and adequate personal protective equipment (PPE) (76, 77) and needs to be equipped with sufficient and appropriate training and supervision for community sensitization activities as well as their own protection for effective service delivery (78, 79). Availability of PPE and adequate training can make the CHWs more confident of delivering the services (80, 81). However, it should be noted that there is scarce of PPE resources in some communities and they need to be appropriately used (82). Of course, the psychosocial support provided by CHWs needs to be monitored by debriefing with supervisors (71). It is also important that CHWs are offered psychosocial support, non-performance-based allowances, additional transport allowances, childcare support and so forth to ensure their active participation and reduced stigmatization and isolation (83). Safety from any potential violence in the community also need to be ensured when home visits are being done by CHWs.

## Policy implications

Despite the high prevalence of mental health disorders in LMICs, compared to high-income countries, it has received fairly little attention in many LMICs (84). Recognizing the high burden of mental health disorders, various LMICs have passed policies and laws, but the implementation of these seems challenging because of fragility of the health systems, inadequate human resources for mental health, and ineffective decision making by health leaders (85). As the COVID-19 has aggravated mental health conditions, it is very important that decision makers and implementors consider the importance of engaging CHWs into the policy discourse. More importantly, integrating mental health services into primary health care through involving and engaging CHWs can help extend the capacity of the workforce to address mental health issues in LMICs. A mixed funding strategy may be required to achieve this, including recognition that some activities may not be funded (86). It is also important to consider CHW roles in relation to other health workforce and integrate CHW intervention into the general health and community system (87).

## Conclusion

The COVID-19 pandemic has made a negative impact on the mental health in LMICs. As a result, health services are looking for alternative ways to tackle the issue. Employing CHWs, training and support them on mental health issues can be a cost saving and effective approach to provide psychosocial support to the people at the local level in LMICs within their fragile health systems.

## Data Availability Statement

The original contributions presented in the study are included in the article/supplementary material, further inquiries can be directed to the corresponding author/s.

## Author Contributions

SM conceived and designed the study. SM, BH-R, UY, SS, and LR contributed to the first draft of the manuscript. MH commented extensively on the first draft and edited the entire paper. All authors contributed to the article and approved the submitted version.

## Conflict of Interest

The authors declare that the research was conducted in the absence of any commercial or financial relationships that could be construed as a potential conflict of interest.
